# Correlation Between Messenger RNA Expression and Clinicopathological Features of Breast Cancer: A systematic review

**DOI:** 10.7150/jca.93607

**Published:** 2024-03-31

**Authors:** Xue Hu, Wanying Xing, Wan Wang

**Affiliations:** Department of Breast Surgery, China-Japan Union Hospital of Jilin University, Changchun, 130033, Jilin Province, China.

**Keywords:** messenger RNA, breast cancer, clinicopathologic features

## Abstract

Background: Meta analysis was adopted to investigate the correlation between messenger ribonucleic acid (mRNA) expression and clinicopathological features of breast cancer (BC). Methods: English databases, PubMed, Web of Science, Embase, and The Cochrane Library, etc., were searched using a computer. The time range of retrieval was set to be from the establishment of the database to December 2023. The search terms were set as “mRNA”, “Breast cancer”, “Pathology”, “Clinicopathological characteristics”, etc. The literatures were screened in line with the inclusion and exclusion criteria, and the data was extracted for analysis by Revman5.3. Results: Finally, 5 suitable included literatures were selected, including 969 patients. The analysis results were found to reveal a significant association between mRNA expression and BC grading (OR = 0.11, 95% CI = 0.04-0.30, Z = 4.26, *P*<0.0001); a significant correlation was observed between mRNA expression and BC staging (OR = 0.19, 95% CI = 0.05-0.65, Z = 2.65, *P* = 0.008<0.05); no correlation was found between mRNA expression and menstrual status of BC patients (OR = 0.63, 95% CI = 0.22-1.78, Z = 0.88, *P* = 0.38>0.05); a correlation was identified between mRNA expression and tumor size in BC (OR = 0.48, 95% CI = 0.24-0.99, Z = 2.00, *P* = 0.05). In the Discussion section, this study, comprising 10 research studies, aimed to explore the correlation between messenger ribonucleic acid and the clinical pathological features of BC. staging and grading of BC, a certain correlation with tumor size, and no correlation with the menstrual status of BC patients.

## Introduction

Breast cancer (BC) originates from duct and acinar epithelium at all levels of breasts, and gradually progresses from adeno-epithelial hyperplasia and atypical hyperplasia to carcinoma *in situ*, early invasive carcinoma, even invasive carcinoma 1. The majority of malignant breast tumors are malignant epithelial tumors, namely BC, which is one of the most common malignant tumors in females. In recent years, the incidence of BC has been increasing dramatically all over the world 2. Patients with BC usually seek medical treatment because of breast palpation and masses, and some patients suffer from clinical manifestations such as “orange peel sign”, “dimple sign”, and nipple discharge. Cancerous tissue infiltrates the skin and can cause skin ulceration and epidermal nodules. Most BCs show ipsilateral axillary or cervical lymph node metastases 3. In 2020, BC replaced lung cancer to become the first diagnosed cancer in the world. In China, BC becomes the most common cancer in women, ranking the first in morbidity and the fifth in mortality. At present, comprehensive treatment strategies, like radiotherapy, chemotherapy, targeted therapy, and immunotherapy, have highly improved prognosis of BC patients 4. However, the occurrence of BC is a process of accumulation of multiple factors and mutations. The etiology of BC is not clear, and the diagnosis and treatment of BC have not made breakthrough progress.

With the development of modern molecular biology, people gradually realize that BC is a malignant tumor caused by the aberration of multiple genes. Its occurrence and development are the results of the synergistic action of multiple genes and multiple segments. The cognization and understanding of biological behaviors of BC at the molecular level are conducive to its clinical diagnosis and treatment 5. In current clinical and scientific researches, a large amount of messenger ribonucleic acid (mRNA) has been discovered to play a crucial role in cancers 6. They are important in the occurrence and development of cancers through different levels of expression and transcription into corresponding proteins. In disease diagnosis, especially in cancers, whole genome sequencing is a very effective and intuitive means. That is, gene sequencing of individuals of species with known gene sequences and analysis of differential gene expression are conducted to obtain significant differences in gene expression between diseased individuals and normal individuals 7. mRNA is a large category of RNA molecules whose function is to transfer genetic information from DNA to the ribosomes, where amino acid sequences of the gene expression products they carry are generated 8. A large deal of studies has confirmed that RNA is widely involved in basic biological processes of cells, such as cell differentiation, proliferation, growth, migration, and apoptosis, together with occurrence and progression of cancers 9.

Under normal circumstances, mRNA converts the genetic information of genes into proteins through the processes of transcription and translation, maintaining the normal functioning of cells. However, in the occurrence and development of breast cancer, the levels of mRNA within cells are influenced by various regulatory mechanisms, potentially leading to a series of abnormal gene expressions. This includes the overexpression of oncogenes and the suppression of tumor suppressor genes, thereby creating favorable conditions for the proliferation and invasion of cancer cells 10. Furthermore, analyzing the mRNA in patients' breast tissues allows for a more accurate identification and classification of subtypes of breast cancer, providing a more precise basis for personalized treatment 11. The expression profile of mRNA may also serve as a biomarker for the early diagnosis of breast cancer, offering doctors earlier and more reliable indicators of the disease and increasing the success rate of treatment 12. Regarding the potential role of mRNA in breast cancer treatment, current research is exploring the use of mRNA technology to intervene in gene expression. This includes methods such as regulating the expression levels of oncogenes and enhancing the function of tumor suppressor genes, aiming to achieve more precise and effective treatment for breast cancer. This opens up new avenues for developing novel treatment strategies and drugs 13. In summary, the relationship between mRNA and breast cancer is a complex and highly researched area. A thorough understanding of this relationship holds the promise of paving new ways for the early diagnosis, treatment, and prevention of breast cancer, contributing to improving the quality of life for patients and increasing the success rate of treatment.

To further explore the correlation between mRNA and BC, the literatures related to mRNA expression in BC in recent years were strictly screened. The following detailed meta-analysis was conducted to probe into whether there was a certain correlation between mRNA and clinicopathologic features of BC patients. It would bring new enlightenment and deep thinking to the diagnosis and treatment of BC clinically.

## Data and methods

### Literature retrieval

Literature retrieval was made through English databases, including PubMed, Web of Science, Embase, and The Cochrane Library, using a computer. The retrieval time ranged from the establishment of these databases to October 2023. The search terms consisted of “mRNA”, “Breast cancer”, “Pathology”, and “Clinicopathological characteristics”.

### Inclusion and exclusion criteria of literatures

The inclusion criteria were made up of: (1) Literatures had been published about the correlation between mRNA and the incidence of BC, limited to English, with no time limit for publication. (2) protein levels associated with the proliferation and invasive capabilities of breast cancer cells. (3) The experimental objects were BC patients. (4) There were clinicopathologic data related to the occurrence of BC (clinical TNM stage and histopathological grade) in the literatures, and the corresponding data could be completely extracted or combined. (5) If the relevant literatures of the same research project had been published in different journals or at different publication time, the one with the most comprehensive and complete information was selected. Different experiments by the same team were also included. If the data needed were lacking in the literature, the original data could be obtained through data calculation or contacting the author of the original text.

Exclusion criteria consisted of: (1) In the literatures, complete data could not be obtained. (2) Literatures had a repeated publication. (3) When 2 studies from the same institution reported the same target outcome, the report with better quality was included. (4) Studies that only involved BC and had nothing to do with clinicopathological features were excluded. (5) Animal experiments or basic research experiments at cellular level were excluded.

### Evaluation of literatures' quality

2 researchers conducted independent reading of retrieved literatures, and they were required to read the full text and extract related data in the literature. When disagreements or disputes arose, it needed to be resolved via a discussion or with the assistance of a third researcher. Jadad was applied to assess the quality of these included literatures in the following terms. (1) Whether the work was a randomized controlled study? (2) Whether the random method adopted was appropriate? (3) Whether the work was a double-blind test? (4) Whether the double-blind approach was suitable? (5) Whether the patients were lost to follow up or dropped out during the research process, whether the reasons were explained, and whether the work applied an intentionality analytic method? “Yes” scored 1 point and “No” scored 0 point, with 5 points in total. With a total score of <2, the literature was classified as low-quality research; while the literature with a score of >2 was classified as high-quality study.

Cochrane Reviewer's Handbook4.2.5 was also applied to appraise the literature quality. Contents for evaluation included: (1) Whether it was a randomized trial? (2) Whether there was an allocation concealment? (3) Whether blind test was used? (4) Whether result data were complete? (5) Whether there were selective reporting results? (6) Whether there was other more biases?

### Data extraction

2 researchers read the literature independently, to determine whether the preliminarily screened literature was a case-control study or cohort study and whether the data were complete. All the relevant studies meeting inclusion criteria were screened out according to requirements of meta-analysis, and literature quality evaluation was carried out for each work. Repeated reports, literatures with poor quality, and works with too little confidence in reporting were excluded. In line with the established table, data extraction was made; a database was built up to verify these data. If a report was not complete, the author would be contacted for a verification, and it would be excluded as it was confirmed to be unavailable. If the 2 researchers disagreed, it was discussed with a third staff to deal with. After the full text of a study was obtained, the data was extracted. If there were duplicated reports, the latest one was chosen. In this work, the data to be extracted consisted of basic literature information (the title, first author, year of publication, authors' information, and literature source), essential characteristics of research objects (gender, age, research sample size, and baseline comparability), methods in the literature, research scheme design, the intervention measures in experimental and control groups, outcome evaluation indicators, as well as outcome data.

### Analysis of statistics

The data were analyzed applying Revman5.3 provided by Cochrane Collaboration. First, the heterogeneity test was made on these research results, and the test level was *α* = 0.05. Meanwhile, Peto method was applied for heterogeneity analysis of literatures. When I^2^<50%, it was thought that there was not any heterogeneity, then fixed-effect model was adopted to analyze the literature. When I^2^>50%, the literature was considered heterogeneous, and random-effect model was used for analysis. The measurement data results using the same unit of measurement were represented by weighted mean difference; otherwise, standard difference was utilized. The enumeration data results were described by the relative risk. All the results were denoted with 95% confidence interval (CI). After funnel plots were drawn, their publication bias was evaluated by symmetry of the funnel plot as well as degree of literature concentration towards the median line. Sensitivity analysis was also adopted to evaluate the reliability and stability of results.

## Results

### Literature retrieval and overview analysis

**Figure 1:**
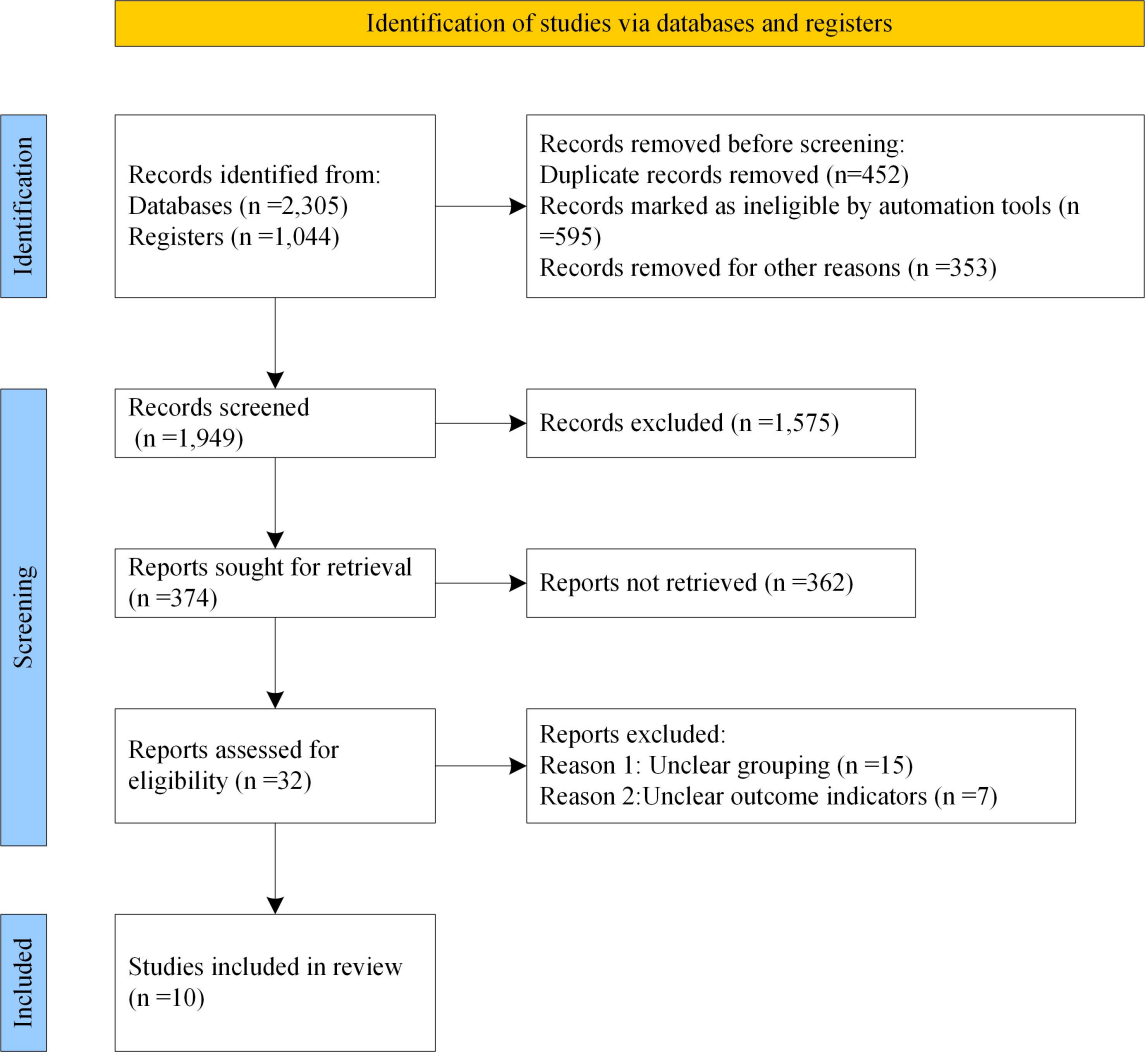
Flow chart of literature retrieval.

3,349 literatures were retrieved in total, of which 1,137 were retrieved by Medline database, 1,100 by EMbase database, 68 by EBSCO database and 1,044 by manual retrieval. On the grounds of inclusion and exclusion criteria, 1,400 literatures initially failed to meet the requirements were excluded. By glancing over the title and abstract, 1,575 literatures clearly did not satisfy the inclusion criteria were also excluded. By reading full text, 362 literatures were eliminated. After careful reading, 22 literatures were then excluded, and 10 literatures meeting the inclusion criteria 14-23 were finally included, as displayed in Figure 1 for detailed screening. The basic information of patients and research indicators in the 5 literatures were listed in Table 1.

### Bias risk assessment of the included literatures

**Figure 2:**
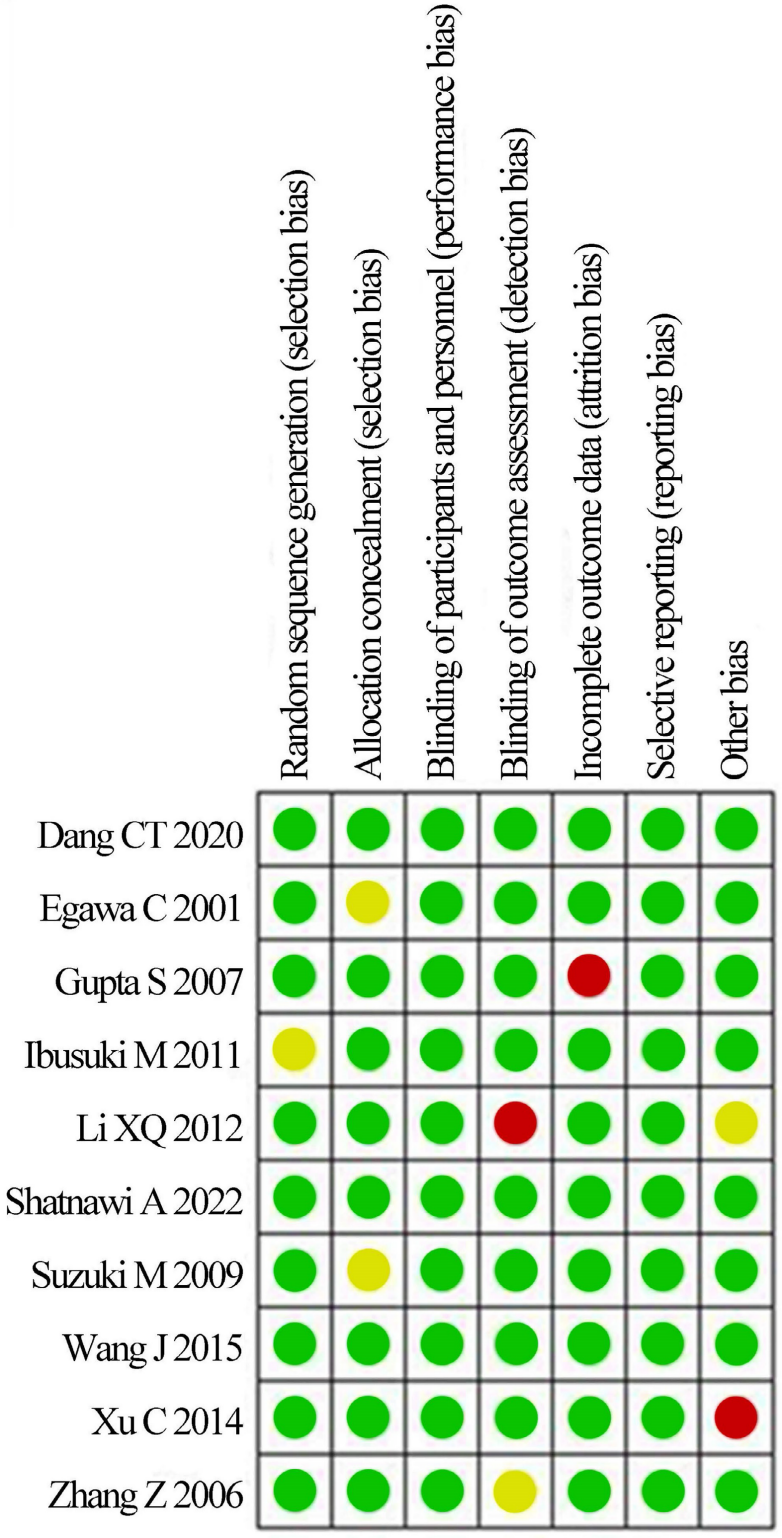
Risk evaluation chart of bias of inclusion literatures.

**Figure 3:**
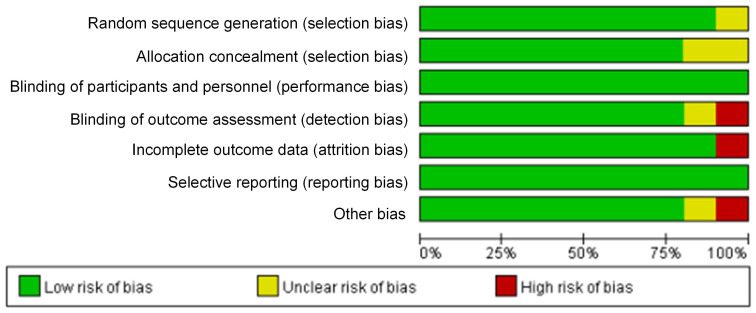
Bar chart of bias risk evaluation for included literatures.

The bias risk assessment tool recommended by *Cochrane Systematic Review Manual* was applied to assess the quality of these included literatures, as results displayed in Figure 2 and Figure 3. There was not any random sequence generation (selection bias), incomplete outcome data (selection bias), or selective reporting (reporting bias) in these 10 studies. On the whole, the risk of included literatures in this work was low.

### Analysis of mRNA expression and BC grading

**Figure 4:**
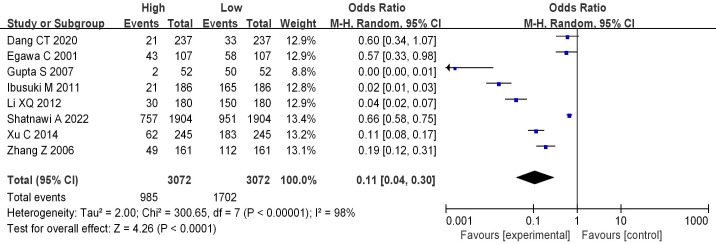
Forest plot of mRNA expression and BC grading.

**Figure 5:**
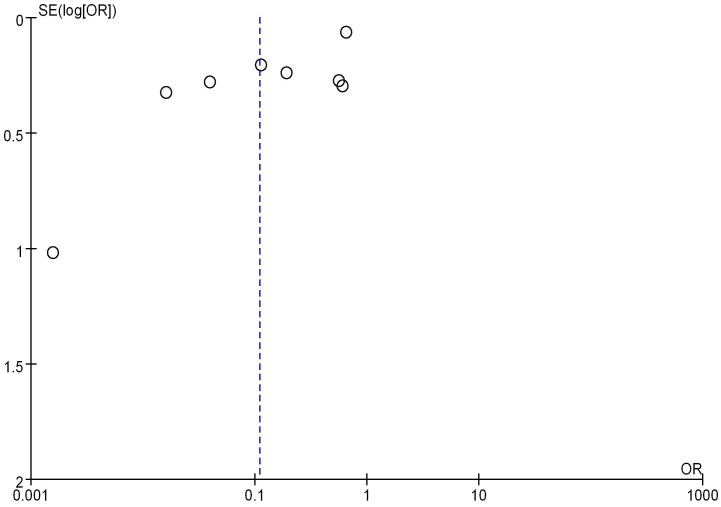
Funnel plot of mRNA and BC grading.

8 literatures included analyzed mRNA expression and BC grading, with the results in Figure 4. Heterogeneity analysis suggested that I^2^ = 98%, *P* < 0.00001, so random-effect model was utilized for subsequent analysis. Odds ratio (OR)=0.11, 95%CI= 0.04-0.30, *Z*= 4.26, and *P* < 0.0001. There were significant differences in mRNA among different BC grades, having the statistical significance (*P*<0.05). The bias analysis results were shown in Figure 5, and the distribution on both sides of the dashed line was asymmetric. Therefore, there was publication bias in the literature.

### Analysis of mRNA expression and BC staging

**Figure 6:**
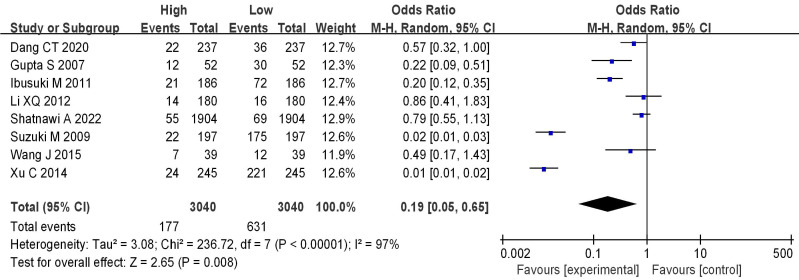
Forest plot of mRNA and BC staging.

**Figure 7:**
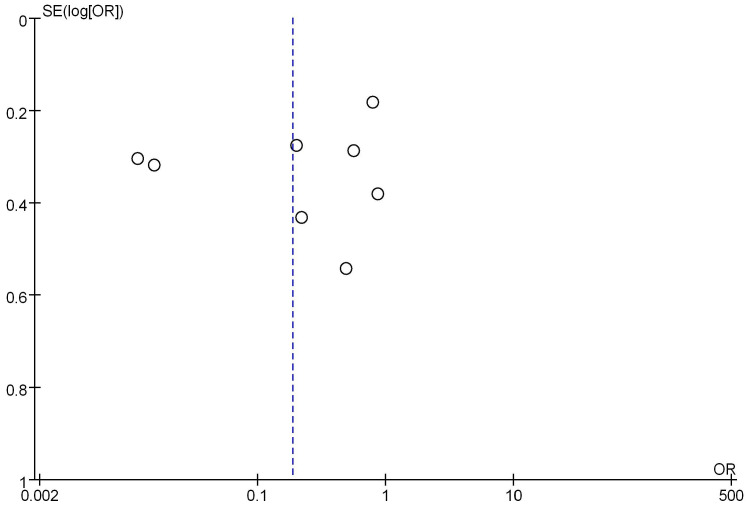
Funnel plot of mRNA expression and BC staging.

8 literatures included analyzed mRNA expression and BC staging, with the results in Figure 6. Heterogeneity analysis showed that I^2^ = 97% and *P* < 0.00001, so random-effect model was adopted for subsequent analysis. The OR=0.19, 95%CI=0.05-0.65, *Z*= 2.65, and *P=*0.008. There were significant differences in mRNA expression among BC in different stages, having statistical significance (*P* < 0.05). The results of the bias analysis (as shown in Figure 7) revealed an asymmetric distribution on both sides of the dashed line, indicating the presence of publication bias in the literature.

### Analysis of mRNA expression and menstrual status

**Figure 8:**
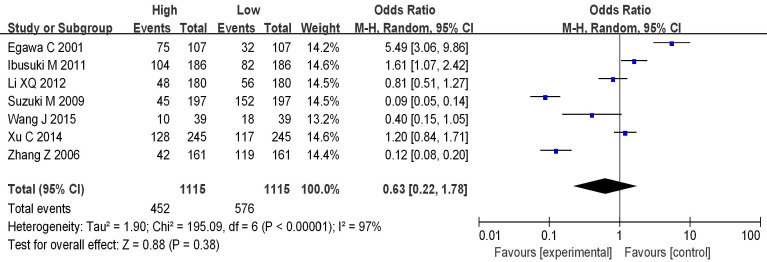
Forest plot of mRNA and menstrual status.

**Figure 9:**
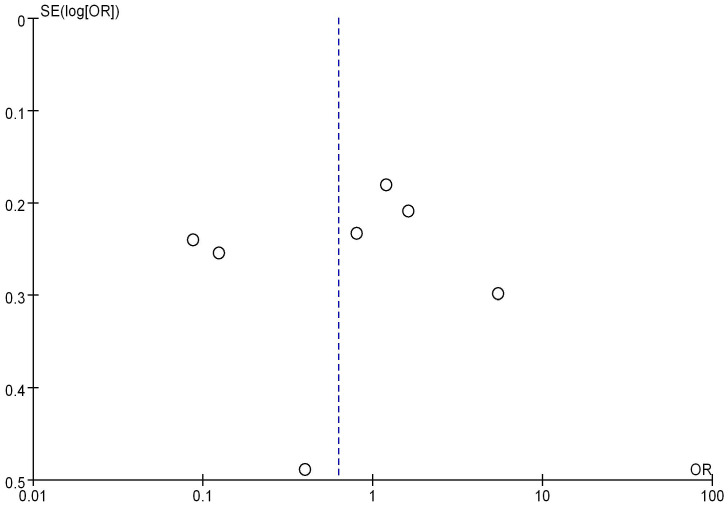
Funnel plot of mRNA expression and menstrual status.

Seven studies analyzed the correlation between mRNA expression and the menstrual status of breast cancer (BC) patients, as shown in Figure 8. Heterogeneity analysis indicated I^2^ = 97%, *P*<0.00001, therefore, a random-effects model was employed for subsequent analysis. The odds ratio (OR) was 0.63, with a 95% CI = of 0.22-1.78, Z=0.88, and *P*=0.38. This suggests no significant correlation between mRNA expression and the menstrual status of BC patients (*P*>0.05). Figure 9 represents a funnel plot for the analysis of mRNA expression and BC patient menstrual status, and the asymmetry on both sides of the dashed line in the plot indicates publication bias in the literature.

### Analysis of mRNA expression and tumor size

**Figure 10:**
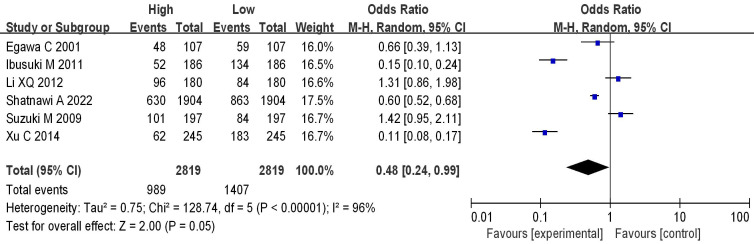
Forest plot of mRNA and BC staging.

**Figure 11:**
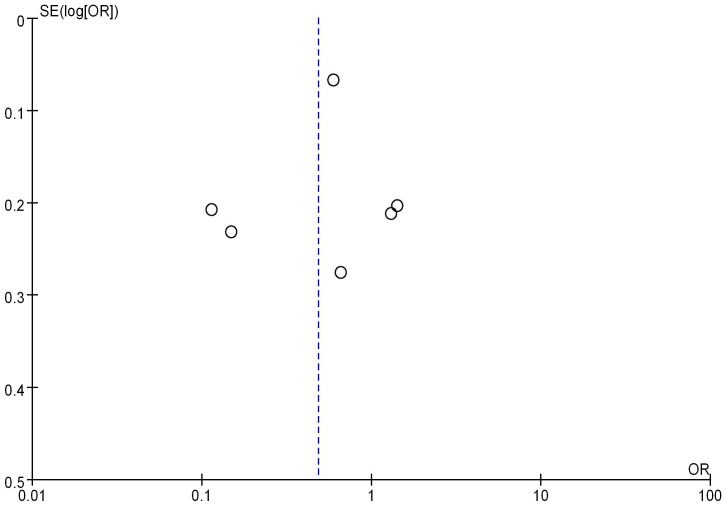
Funnel plot of mRNA expression and BC staging.

Seven studies analyzed the correlation between mRNA expression and the tumor size of BC, as shown in Figure 10. Heterogeneity analysis indicated I^2^ = 96%, *P*<0.00001, therefore, a random-effects model was employed for subsequent analysis. The OR was 0.48, with a 95% CI of 0.24-0.99, Z=2.00, and *P*=0.05. This indicates a correlation between mRNA expression and BC tumor size (*P* = 0.05). The bias analysis results, as shown in Figure 11, demonstrate a relatively small bias in the literature, with the dashed line being roughly symmetrical on both sides.

## Discussion

Approximately 1.7 million people were newly diagnosed with BC worldwide in 2012, and nearly 40,000 female deaths per year are attributed to BC. In 2011, there were 230,480 new BC cases and 39,52,034 deaths due to BC in the United States. In China, the incidence and mortality of BC are also on the rise year by year. As all the facts are presented in front of people's eyes, the physical and mental health of women around the world is seriously affected and their life safety is seriously threatened 24. For a long time, studies about the pathogenesis, clinicopathological features, and prognosis of BC have been increasing by experts and scholars all over the world. In recent years, a series of studies about the correlation between mRNA and BC have been carried out gradually 25.

Histological grading of BC indicates the degree of differentiation of BC, which can be classified into 3 grades. Grade Ⅰ indicates low cellular atypia and high degree of differentiation, with the best prognosis. Grade Ⅱ indicates moderate cellular atypia and moderate differentiation, with a general prognosis; and grade Ⅲ indicates obvious cellular atypia and low differentiation, with a poor prognosis 26. In this work, those below grade Ⅲ were classified as low grade, while grade Ⅲ was sorted as high grade. BC is also clinically classified into stages 0-Ⅳ (TNM staging) according to the tumor size, regional lymph node metastasis, and the distant metastasis 27. In this work, the stages below stage Ⅲ were sorted as low stage while that above stage Ⅲ was taken as high stage. The results indicate a significant correlation between mRNA expression and BC grading (OR=0.11, 95% CI=0.04-0.30, Z=4.26, *P*<0.0001); mRNA expression is also significantly associated with BC staging (OR=0.19, 95% CI=0.05-0.65, Z=2.65, *P*=0.008<0.05). In a study of Zhou et al. (2021) 28, mRNA level was correlated with lymph node metastasis, clinical staging, and histological grading of BC (*P* < 0.05). Zhang et al. (2020) 29 also demonstrated that mRNA had no connection with the patients' age, tumor size, and histological grading of BC, but was correlated with TNM staging, clinical staging, and lymph node metastasis (*P* < 0.001). These findings were all consistent with the results of this work.Additionally, we observed a potential correlation between mRNA expression and BC tumor size (OR=0.48, 95% CI=0.24-0.99, Z=2.00, *P*=0.05). This correlation might be influenced by other factors affecting tumor size, and individual variations may exist. Therefore, the results are not highly significant, and further investigation is needed in the future.Liu et al. (2022) 30 found a significant association between Heterogeneous nuclear ribonucleoprotein C (C1/C2) (HNRNPC) and various malignant features of hepatocellular carcinoma, including tumor size, microvascular infiltration, tumor differentiation, and TNM staging. Similarly, Fu et al. (2023) 31 demonstrated that circTDRD3 is highly expressed in colorectal cancer tissues, positively correlating with overall survival, tumor size, lymph node infiltration, and clinical staging, aligning with the findings in this work. Lastly, we conducted an analysis of the correlation between mRNA expression and the menstrual status of BC patients, revealing no significant association between the two (OR=0.63, 95% CI=0.22-1.78, Z=0.88, *P*=0.38>0.05).

In summary, mRNA expression is significantly correlated with the clinical staging and grading of BC, demonstrating a certain correlation with BC tumor size and no correlation with the menstrual status of BC patients. However, the impact of different mRNA expressions on BC may vary, and individual differences may introduce some biases into the results. Additionally, due to limited data, this work only explored the relationship between mRNA expression and histological grading, clinical TNM staging, tumor size, and menstrual status. In future research, a more comprehensive investigation into the correlation between mRNA expression and the clinical pathology of BC can be pursued.

## Conclusion

In conclusion, this study indicates that mRNA expression is significantly correlated with the clinical staging and grading of BC, demonstrating a certain correlation with BC tumor size and no correlation with the menstrual status of BC patients.However, due to limited data, only mRNA expression, tumor histological grading, clinical TNM staging, tumor size and menstrual statuswere explored in this work. In future studies, the correlation between mRNA expression and clinical pathology of BC could be researched from more aspects.Moreover, some indicators may be influenced by other factors, and the results are not highly significant. Further investigation is needed in the future.

## Figures and Tables

**Table 1:** Basic information of included literatures.

First author	Year of publication	Sample size	Indicators
Li XQ	2012	180	Clinical staging, histological grading, tumor size, and menstrual status
Xu C	2014	245	Clinical staging, histological grading, tumor size, and menstrual status
Zhang Z	2006	161	Histological grading, tumor size
Ibusuki M	2011	186	Clinical staging, histological grading, tumor size, and menstrual status
Suzuki M	2009	197	Clinical staging, tumor size, and menstrual status
Egawa C	2001	107	Histological grading, tumor size, and menstrual status
Shatnawi A	2022	1904	Clinical staging, histological grading, and menstrual status
Wang J	2015	39	Clinical staging, tumor size
Dang CT	2020	237	Clinical staging and histological grading
Gupta S	2007	52	Clinical staging and histological grading
